# Miscellaneous Publications

**Published:** 1852-08

**Authors:** 


*Miscellaneous Publications.*—The following is a list, with occasional brief notices, of a number of publications which have been received during the last two or three months, for which we beg to return our acknowledgments:

*Valedictory Address to the Graduating Class of the Medical College of the State of South Carolina, delivered, by appointment of the Faculty, at the Public Commencement, March* 12*th*, 1852. By E. GEDDINGS, M. D., Professor of Surgery.

*An Introductory Lecture delivered at the opening of the Thirty-second Session of the Medical College of Ohio, October* 15*th*, 1851. By R. D. MUSSEY, M. D., Professor of Surgery in the Medical College of Ohio.

*An Essay on Empirical Remedies; read before the Medical Society of the State of Georgia, April*, 1852. By ROBERT CAMPBELL, M. D.

*First Annual Report of the Board of Trustees of the Williamsburg Dispensary, for the year ending Feb.* 4*th*, 1852. "*To do good, and to communicate, forget not.*"

*Report of the Pennsylvania Hospital for the Insane, for the year* 1851. By THOMAS S. KIRKBRIDE, M. D., Physician to the Institution.

*State of the New York Hospital and Bloomingdale Asylum, for the year* 1851.

*A History of the Art of Midwifery; a Lecture delivered at the College of Physicians and Surgeons, Nov.* 11*th*, 1851, *introductory to a course of private instruction on Operative Midwifery; showing the past inefficiency and present natural incapacity of females in the practice of Obstetrics.* By AUGUSTUS K. GARDNER, M. D., Author of "Old Wine in New Bottles; or, the Spare Hours of a Student in Paris," etc., etc.

This *brochure* gives an interesting historical account of the rise and progress of the science and art of midwifery. The writer, however, is desirous to forestall the decline and fall of this department of medicine, by pointing out the sources of danger. We give the following extract, involving a reflection upon medical education in this branch, which must be admitted to be founded in fact:

"Two dangers now menance the practice of midwifery in this city, either of which threatens to do it an irreparable injury. The one which particularly concerns us is the carelessness and inefficiency of the profession itself. The student at graduation knows little or nothing of the subject. Practically, they are entirely ignorant of its simplest forms. They have no methodised habits, no illustrative reminiscences to throw light upon the obscurities which may occur in their subsequent practice. The student may graduate without ever having attended a single bedside of a parturient woman. After the first few cases, in which he is sufficiently alarmed, but which, fortunately, pass by successfully, he concludes that all the talk of position, presentation, rotation, and such like terms, is all nonsense, or at the best, theoretical, and he joins the "expectant" practitioners, trusts to the *vis medicatrix naturæ*, confidently expects that spontaneous evolution will always occur, and by a dull inactivity, resulting from ignorance, loses the child, and not unfrequently the mother also, and injures the reputation of the profession, the capabilities and attainments of which he does not understand.

"The next great danger springs from the love of mammon. Ignorance and presumption, which thrive upon ignorance and credulity, when 'fools rush in where angels fear to tread.'"

*Amputation of the Entire Lower Jaw, with Disarticulation of both Condyles.* By J. M. Carnochan, M. D., Prof. of the Principles and Operations of Surgery in the New York Medical College, etc., etc.

We confess a lack of admiration for what are frequently called brilliant achievements in operative surgery, while, at the same time, we disclaim, most emphatically, any deficiency of respect for surgical science and the true surgeon. To go a step farther than his predecessors in mutilation, does not seem to us the highest mark of distinction to which the surgeon should aspire. The author of this paper remarks, "To Dupuytren was reserved the glory of having, in 1812, first removed, by a methodical operation, a portion of the body of the inferior maxilla." In what, we would ask, consists the *glory* of having done this? Dupuytren had the boldness to do what others before him had feared to do lest they should place the lives of their patients in too great jeopardy. There may have been merit in the exercise of this boldness, but for the glory, let it rather be reserved for him who decides, in a critical emergency, to spare an important part of the body, and, by assiduous and skillful treatment, preserves life and the body in its native integrity. It is not contended that the operation of Dupuytren required any very superior knowledge or genius for its performance. He *dared* to do it, or his position and reputation were such that he could afford to run the risk of doing it. We know that this kind of distinction is fascinating, and we honestly believe that the influence of this false admiration for operative surgery has rendered that noble department of medicine too often a curse and a disgrace, instead of an honor and a blessing. We have not time nor space to continue our remarks on this subject, but we wish, for one, to be most distinctly on the side of *conservative* surgery. So far from boasting of a bloody, mutilating feat, we think the surgeon in reporting such cases should always feel most solicitous to show abundant reason for the necessity of it, and should write a history of the operations to which he has been reluctantly compelled to resort, in a strain of sadness rather than exultation. Providence deliver ourselves, and the rest of mankind, from the scalpel of the surgeon whose chief ambition is to be a brilliant operator! Far better is it to be at the mercy of the ruffian, for the latter may be content to take our money and spare life.

This strain of remark has been suggested by the title of the paper which is prefixed, but we disclaim any pointed reference to the author of the paper, or to the particular operation which he describes, in what has been said. We have written, as usual, on the spur of the occasion, and should be very sorry to have any personality imputed to us. The feelings we have expressed pertain to abstractions, not to any individual or individuals. God grant that what we have said may have nothing but idealism to rest upon!

In justice to Prof. Carnochan we should state that the terrible operation of removing the entire lower jaw appears to have been skillfully performed. From the history of the case it is charitable to presume an operation to have been proper and necessary; and it resulted in complete success, the patient, ten months afterward, pursuing his vocation as a drayman in a state of perfect health.

It should be added in connection with the notice of this paper, that the award of the credit of the first operation of removing a large portion of the lower jaw to Dupuytren, is called in question by Dr. George C. Blackman, who asserts the honor to be due to Dr. Deadrick, of Rogersville, Tennessee, who performed a similar operation in 1810, and published a report in the '*American Medical Recorder*.' The validity of this claim in behalf of Dr. Deadrick, appears to be fully substantiated by Dr. Blackman, and, indeed, has been admitted by some of the surgical writers in Great Britain.

*The Annual Discourse before the Philadelphia County Medical Society, delivered February* 10, 1852. By the President, Samuel Jackson, M.D., formerly of Northumberland.

A sententious, pithy, excellent discourse.

*Tableaux of New Orleans.* By Bennet Dowler, M. D.

In this memoir, the author evinces that thirst for scientific research for which his contributions to various departments of science have rendered him so distinguished. The curious reader will find in these pages an interesting collection of facts pertaining to the geographical, commercial, geological and sanitary history and relations of the city of New Orleans.

*Contributions to Experimental Physiology.* By Bennet Dowler, M. D.

This is another paper by the same distinguished observer, first issued in the *New Orleans Med. and Surg. Journal*, and republished for distribution in a separate form. Experiments are described in this paper tending to show "That the ligation of the trachea, the divisions of the spinal cord in the cervical and dorsal regions, the removal of the viscera, the destruction of the ganglions and plexuses of the sympathetic nerve, the ligation section and removal of the brachial and ischiatic plexuses, including the nerves of the limbs, do not prevent intelligence, sensation and motions, which are accurate in design, perfect in execution, being simultaneous and altogether voluntary in all parts of the body, even though connected only by muscles." These results are certainly surprising. We shall find space for a condensed account of these experiments in a future number.

*Thirteenth Annual Report of the Directors and Superintendent of the Ohio Lunatic Asylum, to the Fiftieth General Assembly of the State of Ohio, for the year* 1851.

The report by the superintendent, D. S. Hanbury Smith, is a comprehensive, carefully prepared, valuable document.

*The Vital Statistics and Sanitary Condition of Memphis, Tenn. An Anniversary Address, delivered, by appointment, before the Memphis Medical Society, on the 5th of February*, 1852. By Geo. R. Grant, M. D.

If similar investigations and reports could be made in different sections of the country, data would soon accumulate which might lead to valuable inductions.

*Dr. Talcott's Address to the Candidates for the Degree of Doctor in Medicine in the Medical Institution of Yale College, January* 15, 1852.

A sound, sensible discourse.

*Report of the Eastern Lunatic Asylum in the City of Williamsburg, Virginia*, 1851.

This, as well as previous reports by Dr. Galt, the superintendent of the institution, show him to be an able and accomplished physician.

